# Role of NADPH Oxidase-Mediated Reactive Oxygen Species in Podocyte Injury

**DOI:** 10.1155/2013/839761

**Published:** 2013-11-11

**Authors:** Shan Chen, Xian-Fang Meng, Chun Zhang

**Affiliations:** ^1^Department of Nephrology, Union Hospital, Tongji Medical College, Huazhong University of Science and Technology, Wuhan 430022, China; ^2^Department of Neurobiology, Tongji Medical College, Huazhong University of Science and Technology, Wuhan 430030, China

## Abstract

Proteinuria is an independent risk factor for end-stage renal disease (ESRD) (Shankland, 2006). Recent studies highlighted the mechanisms of podocyte injury and implications for potential treatment strategies in proteinuric kidney diseases (Zhang et al., 2012). Reactive oxygen species (ROS) are cellular signals which are closely associated with the development and progression of glomerular sclerosis. NADPH oxidase is a district enzymatic source of cellular ROS production and prominently expressed in podocytes (Zhang et al., 2010). In the last decade, it has become evident that NADPH oxidase-derived ROS overproduction is a key trigger of podocyte injury, such as renin-angiotensin-aldosterone system activation (Whaley-Connell et al., 2006), epithelial-to-mesenchymal transition (Zhang et al., 2011), and inflammatory priming (Abais et al., 2013). This review focuses on the mechanism of NADPH oxidase-mediated ROS in podocyte injury under different pathophysiological conditions. In addition, we also reviewed the therapeutic perspectives of NADPH oxidase in kidney diseases related to podocyte injury.

## 1. Introduction

Chronic kidney disease (CKD) is a major public health problem worldwide. Proteinuria is a common clinical signature and a potent predictor for the progression of CKD. Podocytes are highly differentiated glomerular epithelial cells which lie in the outmost layer of the glomerular filtration barrier. Podocyte foot processes interdigitate with the counterparts of neighboring cells to form the slit diaphragm, which constitutes the final barrier to prevent protein loss from vascular to urinary space. Podocyte injury has been considered as the most important early event initiating glomerulosclerosis in many proteinuric kidney diseases [1]. The slit diaphragm proteins, such as nephrin, podocin, CD2-associated protein (CD2AP), and canonical transient receptor potential-6 channel (TRPC6), have been proved to play key role in maintaining normal podocyte structure and function [2]. Podocyte injury is generally presented as slit diaphragm disruption, actin cytoskeleton rearrangement, podocyte foot processes effacement, and proteinuria.

## 2. Reactive Oxygen Species

Oxidative stress is a characteristic feature in many chronic and inflammatory diseases and indeed associated with the development and progression of CKD [3]. The imbalance between reactive oxygen species (ROS) production and antioxidant systems to scavenge the reactive intermediates will induce oxidative stress. ROS are oxygen-derived small molecules produced as intermediates in the redox processes, such as superoxide (O_2_^•−^), hydroxyl (^•^OH), hypochlorous acid (HOCl), ozone (O_3_), singlet oxygen (^1^O_2_), and hydrogen peroxide (H_2_O_2_) [4]. ROS play important roles in diverse physiological signaling processes but also trigger diseases.

Several oxidoreductases have been identified as potential sources of ROS, including cyclooxygenase, lipoxygenase, cytochrome P450 enzymes, nitric-oxide synthase, xanthine oxidase, mitochondrial NADH: ubiquinone oxidoreductase, and nicotinamide adenine dinucleotide phosphate (NADPH) oxidase. Unlike other enzymes that produce ROS secondary to their specific catalytic process, NADPH oxidase is the only enzyme known to produce ROS as the primary function. And a previous study showed that the primary source of ROS generation in the renal cortex is NADPH oxidase [5].

ROS overproduction was found in many podocyte injury models in vitro and in experimental diseases such as diabetic nephropathy (DN), membranous nephropathy (MN), minimal change disease (MCD), and focal segmental glomerulo-sclerosis (FSGS) [3]. Oxidants in turn cause podocyte injury directly, including DNA damage and apoptosis. However, recent researches showed poor outcomes of antioxidant therapies in clinical studies. It implies that specifically targeting the enzymatic sources of pathophysiologically relevant ROS may be more effective [6].

## 3. NADPH Oxidase and ROS Production

NADPH oxidase is a cytoplasmic enzyme consisting of at least one catalytic, transmembrane-spanning NOX subunit, which produces ROS by transferring electrons from NADPH to molecular oxygen. To date, the NOX family consists of seven members, NOX1–5, and two NOX5-like dual oxidases, Duox1-2. All NOX subunits have been reported to bind one or more regulatory subunits, including p22^phox^, NOXO1 localized in the membrane and cytosolic submits p40^phox^, p47^phox^, p67^phox^, NOXA1, and Rac GTPases. It has long been accepted that the translocation and binding of p47^phox^ with membrane complex of NOX2 and p22^phox^ are the key events leading to the activation of NADPH oxidase and generation of ROS.

The phagocyte NADPH oxidase NOX2, also named gp91^phox^, is the prototypical catalytic subunit of NADPH oxidase. And the backbone of the enzyme is cell membrane-bound cytochrome b558, which consists of NOX2 and p22^phox^. NOX1, NOX3, and NOX4 share some similarities with NOX2, such as structural organization and molecular weight. NOX5 possess an extra aminoterminal calmodulin-like domain that contains four Ca^2+^-binding EF-hands structures. Duox possess an extracellular peroxidase domain that uses the H_2_O_2_ generated by its NOX catalytic moiety in addition to the NOX5-based structure. All the NOX1–4 isoforms require the p22^phox^ subunit, whereas NOX5 and Duox are activated directly by Ca^2+^.

## 4. NADPH Oxidase-Mediated ROS Production and Podocyte Injury

NADPH oxidase plays a fundamental role in maintaining normal cell functions and can be activated by diverse stimuli, such as chemical, physical, environmental, and biological factors. Various NADPH oxidase components are found in the kidney and glomerular cells. NOX1, NOX2, NOX4, p22^phox^, p47^phox^, p67^phox^, and Rac1/2 protein have been indentified within the kidney [7]. Greiber et al. have first demonstrated that in the primary cultured human podocytes, ROS are primarily generated by NADPH oxidases, and the NADPH oxidase subunits p22^phox^, p47^phox^, NOX2, and p67^phox^ were expressed [8]. In addition, Whaley-Connell et al. reported that NOX2, NOX4, p22^phox^, p47^phox^, and Rac1 are expressed immortalized murine podocyte cells [9].

Growing evidence reveals that NADPH oxidase-derived ROS overproduction is importantly involved in the progression of podocyte injury associated with the upregulation of various NOX subtypes [10]. NADPH oxidase in the podocyte is activated by adenosine triphosphate (ATP) [8], chemokine [11], angiotensin II (ANG II) [9], aldosterone [12], high glucose [13–17], C5b-9 [18], advanced oxidation protein products (AOPPs) [29], hyperhomocysteinemia (hHcys) [19–21], insulin [22], puromycin aminonucleoside (PAN) [23, 24], and N-methyl-D-aspartate (NMDA) [25].

### 4.1. Primary Glomerular Nephritis

ROS-mediated glomerular injury was proved in many experimental studies, and ROS may play important roles in the pathogenesis of primary glomerular nephritis [3]. ROS generated from podocytes may trigger podocyte injury. Puromycin aminonucleoside (PAN) was widely used to establish animal models of podocyte injury, such as MCD and FSGS. Incubation of podocytes with puromycin (PAN) induced NOX4 expression and promoted superoxide generation [23, 24]. It led to the upregulation of TRPC6 and a loss of cell viability followed by an elevation in the gap junction protein connexin43 (Cx43) levels. It suggested that NADPH oxidase may promote the cellular structure destruction in podocytes. Known from the Heymann nephritis models of MN in rats, podocytes are the targets of the membrane attack complex C5b-9 [26]. Treatment of immortalized murine podocytes with C5b-9 complex increased ROS production. Immunostaining confirmed the presence of the subunit p47^phox^ in podocytes and showed its translocation to the plasma membrane after C5b-9 assembly [18]. It implies that NADPH oxidase-mediated ROS induced podocyte injury in MN.

### 4.2. Diabetic Nephropathy

It is widely accepted that oxidative stress is a key component in the development of diabetic nephropathy. NADPH oxidases are the main source of ROS in the diabetic kidney. In rat models of streptozotocin-induced diabetic nephropathy, the expression of p47^phox^, p22^phox^, and NOX4 was found upregulated in glomeruli [27]. Podocyte injury has been intensively studied in diabetic nephropathy. Studies using an in vitro model show high glucose-induced NADPH oxidase level and activity elevation, NOX4 upregulation, SOD production, caspase-3 activation, and apoptosis induction in podocyte [13–17, 28]. The accumulation of plasma AOPPs is prevalent in diabetes. Increasing the amount of AOPPs in the media of conditionally immortalized podocytes rapidly triggered the production of intracellular superoxide by activation of NADPH oxidase, and led to podocyte apoptosis [29]. Interestingly, recent studies show that insulin treatment increased the generation of H_2_O_2_, the surface expression of the NADPH oxidase, NOX4, and TRPC6 channels in cultured podocytes [22, 30]. These results suggest that NOX4 may play a role in insulin signaling via TRPC6 channels and may induce insulin resistance in podocytes in diabetic nephropathy.

### 4.3. Hyperhomocysteinemia

Li's team and others have demonstrated that oxidative stress mediated by NADPH oxidase is importantly involved in the progression of glomerular injury associated with hyperhomocysteinemia (hHcys) [31, 32]. Recently, Li et al. found that hHcys induced NOX2 and p47^phox^ expression, NADPH oxidase activation, and superoxide production in immortalized murine podocytes [20, 21, 33, 34]. In addition, hHcys activates lipid raft (LR) clustering which leads to aggregation and assembling of NADPH oxidase subunits to form an active enzyme complex to produce ROS [21].

### 4.4. Others

It was confirmed that NADPH oxidase-derived ROS was induced by NMDA receptor activation in neurons [35], and lately similar results were found in kidney [10]. Recent studies demonstrated that sustained application of NMDA triggered cultured immortalized mouse podocytes produce ROS, associated with increased cell surface expression of p47^phox^ and TRPC6 channels and reduced total and cell surface expression of podocyte slit diaphragm proteins nephrin and podocin without loss of cells [25]. Since NMDA receptor and TRPC6 channels are related to calcium entry in podocytes [36], it is possible that elevation of the intracellular Ca^2+^ concentration may also activate NADPH oxidase in podocytes.

## 5. Role of NADPH Oxidase in Podocyte Injury

### 5.1. Renin-Angiotensin-Aldosterone System

It is noteworthy that podocytes express a functional intrinsic renin-angiotensin-aldosterone system (RAAS) characterized by neprilysin, aminopeptidase A, angiotensin converting enzyme (ACE), prorenin receptor (PRR), renin, and mineralocorticoid receptor (MR), implying a local RAAS in the glomerulus [12, 37]. Previous researches have addressed that ANG II activation of the angiotensin type 1 receptor (AT1R) leads to oxidative stress [38], which is linked to glomerular injury. Moreover, recent evidence indicates that NADPH oxidase was associated with RAAS-induced podocyte and filtration barrier injury [39]. TG (mRen2)27 (Ren2) transgenic rat is a rodent model that overexpresses the mouse rennin gene, exhibits increased RAAS activity, elevated ANG II levels, and oxidative stress [40, 41]. Whaley-Connell et al. demonstrated that increases in kidney cortical tissue ANG II, AT_1_R, and total NADPH oxidase activity submits (NOX2, p67^phox^, p47^phox^, and Rac1) expression, ROS production, podocyte effacement, and loss of the slit-pore diaphragm integrity in Ren2 rats compared with age-matched Sprague-Dawley (S-D) rats [39, 42]. Furthermore, the same team found that direct rennin inhibition, AT_1_R blockade, and MR antagonism could attenuate increased NADPH oxidase activity and submit expression, accompanied by restore podocyte slit diaphragm protein nephrin expression and ultrastructural changes in Ren2 rats [43, 44]. In addition, Fujita's team discovered aldosterone involved podocyte injury [45, 46]. They first explored the presence of MR in podocytes in vivo and in vitro [12]. To study aldosterone-induced podocyte injury, uninephrectomized rats were continuously infused with aldosterone and fed a high-salt diet. It was found that increased p67^phox^, Rac1, NOX2, p47^phox^, and p22^phox^ gene expressions, ROS overproduction, decreased nephrin and podocin expressions, and enhanced expression of aldosterone effector kinase Sgk1 in aldosterone-infused rats could be prevented by treatment of eplerenone, a selective aldosterone receptor blocker [12]. Furthermore, spironolactone, a MR antagonist, inhibited aldosterone induced p67^phox^ translocation and oxidative stress generation in cultured podocytes with consistent expression of MR [12]. Data from these investigations highlight the importance of NADPH oxidase in RAAS-mediated podocyte injury.

### 5.2. Epithelial-to-Mesenchymal Transition

Studies from different laboratories have demonstrated that podocytes could undergo an epithelial-to-mesenchymal transition (EMT) process when challenged by different injurious stimuli, such as transforming growth factor-*β*1 (TGF-*β*1) [47, 48], high glucose [49], adriamycin [47], hcys [19, 20], and mesangial medium from IgA nephropathy patients [50]. Recent studies showed that NOX2 [51], NOX1 [52], NOX4 [53], and Rac1b [54] were related to EMT in different types of epithelial cells, implying that NADPH oxidase-derived ROS may promote activation of the EMT program. Emerging evidence suggests that hHcys induces podocytes to undergo EMT through the activation of NADPH oxidase [19]. EMT in conditionally immortalized mouse podocytes was shown by marked decreases in slit diaphragm-associated protein P-cadherin and zonula occludens-1 (ZO-1) as epithelial markers and by dramatic increases in the expression of mesenchymal markers, fibroblast specific protein-1 and *α*-smooth muscle actin (*α*-SMA). These phenotype changes in podocytes induced by Hcys were accompanied by enhanced superoxide production, which was substantially suppressed by NADPH oxidase inhibition. In wild wildtype (gp91^+/+^) mice, hHcys induced enhanced expression of mesenchymal markers but decreased expression of epithelial markers of podocytes in glomeruli, which were not observed in mice lacking NOX2 (gp91^−/−^) [19]. Recently, Li et al. found that Hcys-induced superoxide production via NADPH oxidase was significantly inhibited by growth hormone (GH), and hcys failed to induce podocyte EMT by GH treatment [20]. These results indicated that NOX-mediated ROS generation is critically involved in mediating podocyte EMT and consequent glomerular functional impairment.

### 5.3. Inflammation

The phagocyte NADPH oxidase NOX2 was initially implicated in innate immunity and inflammation. NOX is recognized as a critical mediator of inflammatory responses in nonimmune systems, such as neuroinflammation, gastric inflammation, colon inflammation, lung inflammation, and vascular inflammation. An anti-inflammatory activity of NOX enzymes, also mentioned in NOX2- and p47^phox^-dificient mice, found that LPS-induced systemic inflammatory responses were significantly enhanced by genetic deficiency of NOX [55]. However, the precise molecular mechanisms accountable for the installation of NADPH oxidase-mediated inflammation are still an unsettled issue. Huber et al. reported that podocytes in culture and podocytes in human kidney sections express a set of chemokine receptors. And chemokine stimulation of the expressed CCRs (CCR4, CCR8, CCR9, and CCR10) and CXCRs (CXCR1, CXCR3, CXCR4, and CXCR5) increased activity of NADPH oxidase and formation of superoxide anions in cultured human podocytes [11]. Moreover, Hu et al. have examined oxidative stress and inflammation in kidney in rats that fed on high fructose, the results showed inflammatory cytokines tumor necrosis factor *α* (TNF-*α*) and interleukin 6 (IL-6); oxidative stress index NOX2 and p22^phox^ were upregulated in kidney, which could be partly restored by farnesoid X receptor (FXR) agonist chenodeoxycholic acid (CDCA) treatment [56]. Induction of proinflammatory cytokine granulocyte macrophage-colony-stimulating factor (GM-CSF) was found in cultured podocytes exposed to ROS [57]. Recent observations from Zhang et al. indicated that NADPH oxidase-derived superoxide production may be an important mechanism mediating hHcys-induced NOD-like receptor protein (NALP3) inflammasome activation in podocytes [58, 59]. Since inflammasome formation and activation may be crucial mechanisms mediating a renal inflammatory response that may directly cause podocyte dysfunction, it is plausible that NADPH oxidase activation is an important early mechanism triggering or promoting podocyte inflammatory injury, leading to glomerularsclerosis.

## 6. Therapeutic Interventions of NADPH Oxidase

### 6.1. NADPH Oxidase Inhibitors

Pharmacological NADPH oxidase inhibitors have been used for many years, such as apocynin, diphenylene iodonium (DPI), and 4-(2-aminoethyl)-benzenesulfonylfluorid (AEBSF). NADPH oxidase inhibition by Apocynin or DPI could attenuate podocyte injury [18, 23, 59, 60]. However, these inhibitors are not specific for NADPH oxidase. Triazolo pyrimidines, represented by the commercially available VAS2870 and its derivative VAS3947, were the first reported selective NADPH oxidase inhibitors [61]. Furthermore, several NADPH oxidase subunit-specific inhibitors have been discovered [62, 63]. Vendrov et al. have shown that GKT136901, a specific inhibitor of NOX1 and NOX4, attenuates ROS generation and atherosclerosis in atherosclerotic lesions [64]. Recently, NOX4 inhibitors with good oral bioavailability were explored for the potential treatment of fibrotic diseases [65]. To date, no evidence shows podocyte protect effects of NADPH oxidase-specific inhibitors. It should be further investigated.

### 6.2. Statins

Statins, also known as 3-Hydroxy-3-methylglutaryl (HMG)-coenzyme A (CoA) reductase inhibitors, exert beneficial actions on oxidative stress independent cholesterol-lowering effects. Statins have an indirect NOX inhibitory action by preventing the isoprenylation of Rac1 [66]. In the Zucker obese rats, a homeostasis model includes assessment-insulin resistance index, rosuvastatin treatment improved albuminuria, podocyte foot process effacement, and NADPH oxidase activity via superoxide dismutase [67]. Moreover, rosuvastatin treatment normalized nephrin expression in Ren2 rats, and attenuated ANG II-dependent increases in NADPH oxidase activity and subunit expression (NOX2, NOX4, Rac1, and p22^phox^) and ROS generation in cultured podocytes [9]. Pretreatment with pitavastatin showed similar podocyte protect effects in Dahl salt-sensitive rats and cultured podocytes via restoring NADPH subunits expression [68].

### 6.3. RAAS Inhibitors

Accumulating evidence reveals that drugs inhibit the RAAS system, including rennin inhibitors, ACE inhibitors (ACEI), AT1R blockers (ARB), aldosterone receptor blocker, and MR antagonist, reduce kidney oxidative stress by the inhibition of NADPH oxidase. Notably, recent study showed that losartan metabolite EXP3179 blocked NADPH oxidase-mediated ROS production [69]. The metabolite may confer to losartan the specific capacity of reduce oxidative stress through NADPH oxidase regulation.

### 6.4. Traditional Chinese Medicine

Triptolide, a major active component of Tripterygium wilfordii Hook F, has potent anti-inflammatory [70] and podocyte protect effects [71]. Recently, Chen et al. have shown that triptolide treatment restored the foot process effacement in PHN rats and inhibited ROS generation and p47^phox^ migration to the podocyte cellular plasma after C5b-9 treatment in vitro [18]. It implyies triptolide interfere with NADPH oxidase activation or expression, thus supports the pleiotropy of the Chinese medicinal herb.

## 7. Conclusions

In conclusion, NADPH oxidase appears to be one of the most promising targets to attenuate podocyte injury. Oxidative stress is a common mechanism of CKD [72]. Restore podocyte injury in the early stage will be helpful to prevent CKD patients progress to ESRD. DATA from experimental models showed that the activation of NADPH oxidase in podocytes could be partly blocked by statins [9, 68], ARB [69], and triptolide [18]. It implies that the protection effects of such drugs in podocytes may include various mechanisms. Furthermore, tissue specific, isoform-selective NADPH oxidase inhibitors will be explored in elucidating the full therapeutic potential of NADPH oxidase in the progression of kidney diseases.

## References

[1] Shankland SJ (2006). The podocyte’s response to injury: role in proteinuria and glomerulosclerosis. *Kidney International*.

[2] Tryggvason K, Patrakka J, Wartiovaara J (2006). Hereditary proteinuria syndromes and mechanisms of proteinuria. *The New England Journal of Medicine*.

[3] Shah SV, Baliga R, Rajapurkar M, Fonseca VA (2007). Oxidants in chronic kidney disease. *Journal of the American Society of Nephrology*.

[4] Bedard K, Krause K-H (2007). The NOX family of ROS-generating NADPH oxidases: physiology and pathophysiology. *Physiological Reviews*.

[5] Wang D, Chen Y, Chabrashvili T (2003). Role of oxidative stress in endothelial dysfunction and enhanced responses to angiotensin II of afferent arterioles from rabbits infused with angiotensin II. *Journal of the American Society of Nephrology*.

[6] Altenhofer S, Kleikers PW, Radermacher KA (2012). The NOX toolbox: validating the role of NADPH oxidases in physiology and disease. *Cellular and Molecular Life Sciences*.

[7] Modlinger P, Chabrashvili T, Gill PS (2006). RNA silencing in vivo reveals role of p22^phox^ in rat angiotensin slow pressor response. *Hypertension*.

[8] Greiber S, Münzel T, Kästner S, Müller B, Schollmeyer P, Pavenstädt H (1998). NAD(P)H oxidase activity in cultured human podocytes: effects of adenosine triphosphate. *Kidney International*.

[9] Whaley-Connell A, Habibi J, Nistala R (2008). Attenuation of NADPH oxidase activation and glomerular filtration barrier remodeling with statin treatment. *Hypertension*.

[10] Zhang C, Yi F, Xia M (2010). NMDA receptor-mediated activation of NADPH oxidase and glomerulosclerosis in hyperhomocysteinemic rats. *Antioxidants and Redox Signaling*.

[11] Huber TB, Reinhardt HC, Exner M (2002). Expression of functional CCR and CXCR chemokine receptors in podocytes. *Journal of Immunology*.

[12] Shibata S, Nagase M, Yoshida S, Kawachi H, Fujita T (2007). Podocyte as the target for aldosterone: roles of oxidative stress and Sgk1. *Hypertension*.

[13] Xu J, Li Z, Xu P, Yang Z (2012). Protective effects of leukemia inhibitory factor against oxidative stress during high glucose-induced apoptosis in podocytes. *Cell Stress Chaperones*.

[14] Toyonaga J, Tsuruya K, Ikeda H (2011). Spironolactone inhibits hyperglycemia-induced podocyte injury by attenuating ROS production. *Nephrology Dialysis Transplantation*.

[15] Eid AA, Ford BM, Block K (2010). AMP-activated Protein Kinase (AMPK) negatively regulates Nox4-dependent activation of p53 and epithelial cell apoptosis in diabetes. *The Journal of Biological Chemistry*.

[16] Piwkowska A, Rogacka D, Jankowski M, Dominiczak MH, Stepiński JK, Angielski S (2010). Metformin induces suppression of NAD(P)H oxidase activity in podocytes. *Biochemical and Biophysical Research Communications*.

[17] Susztak K, Raff AC, Schiffer M, Böttinger EP (2006). Glucose-induced reactive oxygen species cause apoptosis of podocytes and podocyte depletion at the onset of diabetic nephropathy. *Diabetes*.

[18] Chen Z-H, Qin W-S, Zeng C-H (2010). Triptolide reduces proteinuria in experimental membranous nephropathy and protects against C5b-9-induced podocyte injury in vitro. *Kidney International*.

[19] Zhang C, Xia M, Boini KM (2011). Epithelial-to-mesenchymal transition in podocytes mediated by activation of NADPH oxidase in hyperhomocysteinemia. *Pflügers Archiv—European Journal of Physiology*.

[20] Li C-X, Xia M, Han W-Q (2011). Reversal by growth hormone of homocysteine-induced epithelial-to- mesenchymal transition through membrane raft-redox signaling in podocytes. *Cellular Physiology and Biochemistry*.

[21] Zhang C, Hu J-J, Xia M, Boini KM, Brimson C, Li P-L (2010). Redox signaling via lipid raft clustering in homocysteine-induced injury of podocytes. *Biochimica et Biophysica Acta*.

[22] Kim EY, Anderson M, Dryer SE (2012). Insulin increases surface expression of TRPC6 channels in podocytes: role of NADPH oxidases and reactive oxygen species. *American Journal of Physiology—Renal Physiology*.

[23] Yan Q, Gao K, Chi Y (2012). NADPH oxidase-mediated upregulation of connexin43 contributes to podocyte injury. *Free Radical Biology and Medicine*.

[24] Wang Z, Wei X, Zhang Y (2009). NADPH oxidase-derived ROS contributes to upregulation of TRPC6 expression in puromycin aminonucleoside-induced podocyte injury. *Cellular Physiology and Biochemistry*.

[25] Kim EY, Anderson M, Dryer SE (2012). Sustained activation of N-methyl-D-aspartate receptors in podoctyes leads to oxidative stress, mobilization of transient receptor potential canonical 6 channels, nuclear factor of activated T cells activation, and apoptotic cell death. *Molecular Pharmacology*.

[26] Ponticelli C (2007). Membranous nephropathy. *Journal of Nephrology*.

[27] Etoh T, Inoguchi T, Kakimoto M (2003). Increased expression of NAD(P)H oxidase subunits, NOX4 and p22^phox^, in the kidney of streptozotocin-induced diabetic rats and its reversibity by interventive insulin treatment. *Diabetologia*.

[28] Eid AA, Gorin Y, Fagg BM (2009). Mechanisms of podocyte injury in diabetes role of cytochrome P450 and NADPH oxidases. *Diabetes*.

[29] Zhou LL, Hou FF, Wang GB (2009). Accumulation of advanced oxidation protein products induces podocyte apoptosis and deletion through NADPH-dependent mechanisms. *Kidney International*.

[30] Johnson AM, Olefsky JM (2013). The origins and drivers of insulin resistance. *Cell*.

[31] Yi F, Xia M, Li N, Zhang C, Tang L, Li P-L (2009). Contribution of guanine nucleotide exchange factor Vav2 to hyperhomocysteinemic glomerulosclerosis in rats. *Hypertension*.

[32] Yang Z-Z, Zou A-P (2003). Homocysteine enhances TIMP-1 expression and cell proliferation associated with NADH oxidase in rat mesangial cells. *Kidney International*.

[33] Zhang C, Hu J-J, Xia M (2010). Protection of podocytes from hyperhomocysteinemia-induced injury by deletion of the gp91^phox^ gene. *Free Radical Biology and Medicine*.

[34] Boini KM, Xia M, Li C (2011). Acid sphingomyelinase gene deficiency ameliorates the hyperhomocysteinemia- induced glomerular injury in mice. *American Journal of Pathology*.

[35] Brennan AM, Suh SW, Won SJ (2009). NADPH oxidase is the primary source of superoxide induced by NMDA receptor activation. *Nature Neuroscience*.

[36] Chen S, He F-F, Wang H (2011). Calcium entry via TRPC6 mediates albumin overload-induced endoplasmic reticulum stress and apoptosis in podocytes. *Cell Calcium*.

[37] Velez JCQ, Bland AM, Arthur JM, Raymond JR, Janech MG (2007). Characterization of renin-angiotensin system enzyme activities in cultured mouse podocytes. *American Journal of Physiology—Renal Physiology*.

[38] Griendling KK, Ushio-Fukai M (2000). Reactive oxygen species as mediators of angiotensin II signaling. *Regulatory Peptides*.

[39] Whaley-Connell AT, Chowdhury NA, Hayden MR (2006). Oxidative stress and glomerular filtration barrier injury: role of the renin-angiotensin system in the Ren2 transgenic rat. *American Journal of Physiology—Renal Physiology*.

[40] Blendea MC, Jacobs D, Stump CS (2005). Abrogation of oxidative stress improves insulin sensitivity in the Ren-2 rat model of tissue angiotensin II overexpression. *American Journal of Physiology—Endocrinology and Metabolism*.

[41] Campbell DJ, Rong P, Kladis A, Rees B, Ganten D, Skinner SL (1995). Angiotensin and bradykinin peptides in the TGR(mRen-2)27 rat. *Hypertension*.

[42] Whaley-Connell A, Habibi J, Johnson M (2009). Nebivolol reduces proteinuria and renal NADPH oxidase-generated reactive oxygen species in the transgenic Ren2 rat. *American Journal of Nephrology*.

[43] Whaley-Connell A, Nistala R, Habibi J (2010). Comparative effect of direct renin inhibition and AT1R blockade on glomerular filtration barrier injury in the transgenic Ren2 rat. *American Journal of Physiology—Renal Physiology*.

[44] Whaley-Connell A, Habibi J, Wei Y (2009). Mineralocorticoid receptor antagonism attenuates glomerular filtration barrier remodeling in the transgenic Ren2 rat. *American Journal of Physiology—Renal Physiology*.

[45] Nagase M, Matsui H, Shibata S, Gotoda T, Fujita T (2007). Salt-induced nephropathy in obese spontaneously hypertensive rats via paradoxical activation of the mineralocorticoid receptor: role of oxidative stress. *Hypertension*.

[46] Nagase M, Fujita T (2008). Aldosterone and glomerular podocyte injury. *Clinical and Experimental Nephrology*.

[47] Kang YS, Li Y, Dai C, Kiss LP, Wu C, Liu Y (2010). Inhibition of integrin-linked kinase blocks podocyte epithelial-mesenchymal transition and ameliorates proteinuria. *Kidney International*.

[48] Sam R, Wanna L, Gudehithlu KP (2006). Glomerular epithelial cells transform to myofibroblasts: early but not late removal of TGF-*β*1 reverses transformation. *Translational Research*.

[49] Dai H-Y, Zheng M, Lv L-L (2012). The roles of connective tissue growth factor and integrin-linked kinase in high glucose-induced phenotypic alterations of podocytes. *Journal of Cellular Biochemistry*.

[50] Wang C, Liu X, Ke Z (2012). Mesangial medium from IgA nephropathy patients induces podocyte epithelial-to-mesenchymal transition through activation of the phosphatidyl inositol-3-kinase/Akt signaling pathway. *Cellular Physiology and Biochemistry*.

[51] Djamali A, Reese S, Hafez O (2012). Nox2 is a mediator of chronic CsA nephrotoxicity. *American Journal of Transplantation*.

[52] Liu F, Gomez Garcia AM, Meyskens FL (2012). NADPH oxidase 1 overexpression enhances invasion via matrix metalloproteinase-2 and epithelial-mesenchymal transition in melanoma cells. *Journal of Investigative Dermatology*.

[53] Boudreau HE, Casterline BW, Rada B, Korzeniowska A, Leto TL (2012). Nox4 involvement in TGF-beta and SMAD3-driven induction of the epithelial-to-mesenchymal transition and migration of breast epithelial cells. *Free Radical Biology and Medicine*.

[54] Lee K, Chen QK, Lui C, Cichon MA, Radisky DC, Nelson CM (2012). Matrix compliance regulates Rac1b localization, NADPH oxidase assembly, and epithelial-mesenchymal transition. *Molecular Biology of the Cell*.

[55] Zhang W-J, Wei H, Frei B (2009). Genetic deficiency of NADPH oxidase does not diminish, but rather enhances, LPS-induced acute inflammatory responses in vivo. *Free Radical Biology and Medicine*.

[56] Hu Z, Ren L, Wang C, Liu B, Song G (2012). Effect of chenodeoxycholic acid on fibrosis, inflammation and oxidative stress in kidney in high-fructose-fed Wistar rats. *Kidney & Blood Pressure Research*.

[57] Greiber S, Müller B, Daemisch P, Pavenstädt H (2002). Reactive oxygen species alter gene expression in podocytes: induction of granulocyte macrophage-colony-stimulating factor. *Journal of the American Society of Nephrology*.

[58] Zhang C, Boini KM, Xia M (2012). Activation of Nod-like receptor protein 3 inflammasomes turns on podocyte injury and glomerular sclerosis in hyperhomocysteinemia. *Hypertension*.

[59] Abais JM, Zhang C, Xia M (2013). NADPH oxidase-mediated triggering of inflammasome activation in mouse podocytes and glomeruli during hyperhomocysteinemia. *Antioxidants & Redox Signaling*.

[60] Kinugasa S, Tojo A, Sakai T (2011). Selective albuminuria via podocyte albumin transport in puromycin nephrotic rats is attenuated by an inhibitor of NADPH oxidase. *Kidney International*.

[61] Wind S, Beuerlein K, Eucker T (2010). Comparative pharmacology of chemically distinct NADPH oxidase inhibitors. *British Journal of Pharmacology*.

[62] Gianni D, Taulet N, Zhang H (2010). A novel and specific NADPH oxidase-1 (Nox1) small-molecule inhibitor blocks the formation of functional invadopodia in human colon cancer cells. *ACS Chemical Biology*.

[63] Bhandarkar SS, Jaconi M, Fried LE (2009). Fulvene-5 potently inhibits NADPH oxidase 4 and blocks the growth of endothelial tumors in mice. *The Journal of Clinical Investigation*.

[64] Vendrov AE, Madamanchi NR, Niu X-L (2010). NADPH oxidases regulate CD44 and hyaluronic acid expression in thrombin-treated vascular smooth muscle cells and in atherosclerosis. *The Journal of Biological Chemistry*.

[65] Laleu B, Gaggini F, Orchard M (2010). First in class, potent, and orally bioavailable NADPH oxidase isoform 4 (Nox4) inhibitors for the treatment of idiopathic pulmonary fibrosis. *Journal of Medicinal Chemistry*.

[66] Manea A (2010). NADPH oxidase-derived reactive oxygen species: involvement in vascular physiology and pathology. *Cell and Tissue Research*.

[67] Whaley-Connell A, Demarco VG, Lastra G (2007). Insulin resistance, oxidative stress, and podocyte injury: role of rosuvastatin modulation of filtration barrier injury. *American Journal of Nephrology*.

[68] Cheng XW, Kuzuya M, Sasaki T (2011). Inhibition of mineralocorticoid receptor is a renoprotective effect of the 3-hydroxy-3-methylglutaryl-coenzyme A reductase inhibitor pitavastatin. *Journal of Hypertension*.

[69] Fortuno A, Bidegain J, Robador PA (2009). Losartan metabolite EXP3179 blocks NADPH oxidase-mediated superoxide production by inhibiting protein kinase C: potential clinical implications in hypertension. *Hypertension*.

[70] Qui D, Kao PN (2003). Immunosuppressive and anti-inflammatory mechanisms of triptolide, the principal active diterpenoid from the Chinese medicinal herb Tripterygium wilfordii Hook. f. *Drugs in R and D*.

[71] Zheng C-X, Chen Z-H, Zeng C-H, Qin W-S, Li L-S, Liu Z-H (2008). Triptolide protects podocytes from puromycin aminonucleoside induced injury in vivo and in vitro. *Kidney International*.

[72] Vaziri ND (2004). Roles of oxidative stress and antioxidant therapy in chronic kidney disease and hypertension. *Current Opinion in Nephrology and Hypertension*.

